# Uremic Toxins, Oxidative Stress, Atherosclerosis in Chronic Kidney Disease, and Kidney Transplantation

**DOI:** 10.1155/2021/6651367

**Published:** 2021-02-11

**Authors:** Ewa Wojtaszek, Urszula Oldakowska-Jedynak, Marlena Kwiatkowska, Tomasz Glogowski, Jolanta Malyszko

**Affiliations:** Department of Nephrology, Dialysis & Internal Diseases, The Medical University of Warsaw, Poland

## Abstract

Patients with chronic kidney disease (CKD) are at a high risk for cardiovascular disease (CVD), and approximately half of all deaths among patients with CKD are a direct result of CVD. The premature cardiovascular disease extends from mild to moderate CKD stages, and the severity of CVD and the risk of death increase with a decline in kidney function. Successful kidney transplantation significantly decreases the risk of death relative to long-term dialysis treatment; nevertheless, the prevalence of CVD remains high and is responsible for approximately 20-35% of mortality in renal transplant recipients. The prevalence of traditional and nontraditional risk factors for CVD is higher in patients with CKD and transplant recipients compared with the general population; however, it can only partly explain the highly increased cardiovascular burden in CKD patients. Nontraditional risk factors, unique to CKD patients, include proteinuria, disturbed calcium, and phosphate metabolism, anemia, fluid overload, and accumulation of uremic toxins. This accumulation of uremic toxins is associated with systemic alterations including inflammation and oxidative stress which are considered crucial in CKD progression and CKD-related CVD. Kidney transplantation can mitigate the impact of some of these nontraditional factors, but they typically persist to some degree following transplantation. Taking into consideration the scarcity of data on uremic waste products, oxidative stress, and their relation to atherosclerosis in renal transplantation, in the review, we discussed the impact of uremic toxins on vascular dysfunction in CKD patients and kidney transplant recipients. Special attention was paid to the role of native and transplanted kidney function.

## 1. Introduction

Patients with chronic kidney disease (CKD) are at a high risk for cardiovascular disease (CVD), and approximately half of all deaths among patients with CKD are a direct result of CVD. Premature cardiovascular disease extends from mild to moderate stages of CKD, and the severity of CVD and the risk of death increase with a decline in kidney function [1–3].

Moreover, the nature and spectrum of cardiovascular disease in CKD are recognized to be different from that in people without kidney disease including atherosclerosis, arteriosclerosis, calcific arterial and valve disease, left ventricular remodeling and dysfunction, arrhythmia, and sudden cardiac death.

Successful kidney transplantation significantly decreases the risk of death relative to long-term dialysis treatment [4]. Nevertheless, the prevalence of cardiovascular disease in this population is high and is responsible for approximately 20-35% of mortality in renal transplant recipients [5].

The prevalence of traditional and nontraditional riskfactors for CVD is higher in patients with CKD compared with the general population; however, it can only partly explain such sorely increased cardiovascular burden in CKD patients [2, 6]. Nontraditional risk factors, unique to CKD patients, include proteinuria, disturbed calcium and phosphate metabolism, anemia, fluid overload, and accumulation of uremic toxins. This accumulation of uremic toxins is associated with systemic alterations including inflammation and oxidative stress which are considered crucial in the progression of CKD-related CVD.

Kidney transplantation can mitigate the impact of some of these nontraditional risk factors, but they typically persist to some degree following transplantation. The restoration of renal function favorably modifies cardiovascular risk in transplant recipients, and each 5 ml/min/1.73 m^2^ increase in eGFR is associated with a 15% reduction in cardiovascular disease and mortality [7]. However, some specific for this population factors, such as immune activation and immunosuppressant agents, may be involved in the increased cardiovascular risk of cardiovascular disease [5].

## 2. Uremic Toxins

The progressive loss of kidney function is accompanied by the retention of plenty of metabolites, due to a decrease in their renal clearance and/or a rise in production. Many of these solutes have been shown to exert biological activity, thereby affecting the functioning of cells and affecting metabolic processes, resulting in the uremic syndrome. Generally, they may originate from endogenous metabolism, be produced by microbial metabolism, or be ingested from an endogenous source. According to the European Uremic Toxin Work Group (EUtox) organic uremic toxins are classified according to their physicochemical properties and possibilities of removal by dialysis [8]:

1. Small, water-soluble molecules with a maximum molecular weight (MW) of 500 Da which can be easily removed by dialysis; molecules in this group include, i.a., guanidines (asymmetric dimethylarginine (ADMA) and symmetric dimethylarginine (SDMA)), oxalate, methylamines (trimethylamine-N-oxide (TMAO)), polyamines, urea, carbamylated compounds, and purines
2. Middle molecules—small proteins or peptides with MW ≥ 500 Da, although most of them have MW > 10000 Da. They are often expressed in response to other toxins (e.g., cytokines), and their concentration depends both from retention and on endocrine and paracrine mechanisms. Dialytic removal of middle molecules is possible with membranes with a large enough pore size used in either diffusive or convective mode. Compounds in this group include angiogenin, atrial natriuretic peptide (ANP), *β*_2_-microglobulin, complement factors D and Ba, cytokines (IL-6, IL-18, IL-1*β*, and TNF*α*), endothelin, fibroblast growth factor-23 (FGF-23), modified lipids and lipoproteins, pentraxin-3, VEGF, and parathyroid hormone
3. Protein-bound molecules—the heterogeneous group of generally low MW solutes, which due to their protein binding are difficult to remove by dialysis; many of these molecules are generated by the intestine microbiota; the main compounds in this group are advanced glycation end products (AGEs), cresols (p-cresyl sulfate, p-cresyl glucuronide), hippurates, homocysteine, indoles (indoxyl sulfate, indole acetic acid), kynurenines, and phenols (phenylacetic acid) [8]

Accumulating data suggest that uremic toxins contribute substantially to the development and severity of cardiovascular disease in CKD patients.  Table 1 summarizes the mechanisms of action of selected uremic toxin impact on cardiovascular damage.

## 3. Atherosclerosis in Chronic Kidney Disease

Accumulating data suggest that atherosclerosis starts from early stages of CKD and remaining high as CKD progresses [33]. CKD-related endothelial dysfunction plays an important role in the development of atherosclerosis [34, 35]. It is characterized by increased oxidative stress, expression of proinflammatory and prothrombotic molecules, and decreased capabilities of endothelial repair. Uremic toxins can contribute to these deleterious effects on the endothelium [36–38]. There is a correlation between inflammation, oxidative stress, endothelial dysfunction, and markers of vasculopathy and kidney function [39–41].

**Figure 1:**
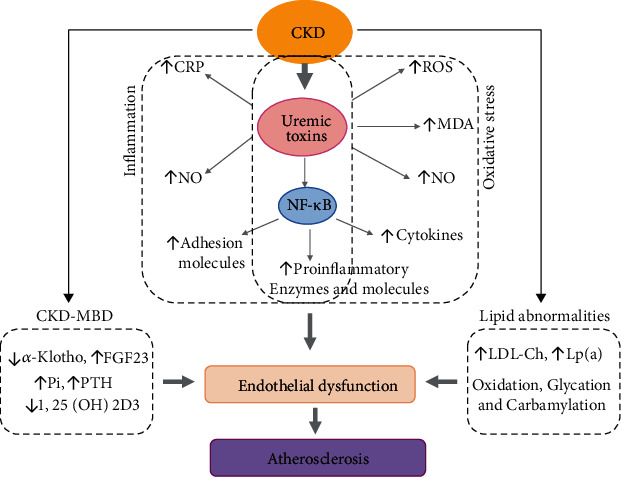
Factors potentially contributing to atherosclerosis in CKD. CRP: C-reactive protein; NO: nitric oxide; ROS: reactive oxygen species; MDA: malondialdehyde; NF-*κ*B: nuclear factor kappa-light-chain-enhancer of activated B cells; FGF23: fibroblast growth factor 23; Pi: phosphates; PTH: parathyroid hormone; 1,25(OH)2D3: 1,25-dihydroxyvitamin D3; LDL: low-density lipoprotein; Lp(a): lipoprotein a; CKD: chronic kidney disease; CKD-MBD: chronic kidney disease-mineral bone disorder.

The vascular toxicity of uremic toxins has been demonstrated in clinical studies among chronic kidney disease, dialysis, and kidney transplant patients. Decreased kidney function impacts the levels of these solutes and may be a relevant confounder when the association between uremic toxins and hard cardiovascular outcomes is studied. The factors potentially contributing to atherosclerosis in CKD patients are presented in Figure 1.

## 4. Uremic Toxins and Kidney Function

### 4.1. Protein-Bound Uremic Toxins

Protein-bound uremic toxins (pbUTs)—p-cresyl sulfate (p-CS), p-cresyl glucuronide (p-CG), indoxyl sulfate (IxS), and indole-3-acetic acid (IAA)—originate from the metabolism of the intestinal microbiota of aromatic amino acids (tyrosine, phenylalanine, and tryptophan) [42–44]. In the distal colon segment, tryptophan is converted into indole and IAA, and tyrosine and phenylalanine into p-cresol. In the colon mucosa and liver, p-cresol is partly detoxified into p-CS and p-CG, and indole into IxS [42–44]. In blood, pbUTs bind on serum albumin [45] are removed by the kidneys—free fraction by glomerular filtration and protein-bound via tubular secretion [43, 44].

The serum levels of pbUTs are inversely related to renal function, and the serum concentrations increase progressively with the progression of CKD in adults and pediatric CKD patients [44, 46–51]. It was demonstrated that free and total fractions of toxins increase progressively from early stages of CKD with significantly higher concentrations in later stages [44, 46–48, 51]. Total and free fractions of p-CS and IxS correlate inversely with eGFR [46–48] and are comparable in patients on peritoneal dialysis and hemodialysis [48]. In dialyzed patients, residual renal function substantially contributes to uremic toxin levels both in patients on maintenance hemodialysis and peritoneal dialysis [52, 53]. Together with the loss of kidney function serum concentrations, there is a rise in uremic toxin levels [52, 53].

Few studies evaluated the levels of pbUTs in transplanted patients [51, 54–56]. In prospective studies by Liaeuf et al. [51, 55] and Poesen et al. [54], it was demonstrated that serum levels of IxS, IAA, and p-CS decreased significantly within a few days and then remained stable during 12 months after transplantation. Moreover, the levels of pbUTs in transplanted subjects were even lower than in controls with comparable kidney function. The cause of this phenomenon remains unclear. The possible explanations of these findings are the changes in gut microbiota after transplantation and the impact of immunosuppressant agents and antibiotics [57].

### 4.2. Asymmetric Dimethylarginine (ADMA) and Symmetric Dimethylarginine (SDMA)

Serum levels of ADMA and SDMA are elevated in patients with CKD [58, 59]. For SDMA, renal excretion is the major pathway of elimination, and SDMA levels are closely related to eGFR. The kidneys also play a central role in the elimination of ADMA; however, the removal of ADMA takes place both by excretion in the urine and by degradation by dimethylarginine dimethylaminohydrolase (DDAH) and transamination by alanine glyoxylate aminotransferase 2 (AGXT2), enzymes primarily expressed in the kidneys. This may explain why in patients with autosomal dominant polycystic kidney disease or kidney diseases with proteinuria, ADMA levels arise earlier and are highly independent on eGFR [60].

The data on levels of ADMA and SDMA in renal transplant patients are scarce and somewhat inconsistent. Most often, plasma ADMA levels demonstrated a biphasic course after successful kidney transplantation with a transient rise in the immediate postoperative period followed by a subtle decline in the weeks; however, the change did not correlate with improvement of graft function. ADMA levels remained elevated compared with CKD patients, matched for age and comorbidities [61–64]. A potential explanation of the increase of ADMA levels in the posttransplant period may be the effect of methylarginine release triggered by surgery, ischemia/reperfusion injury, and the catabolic effect of corticosteroids [61, 64, 65]. The persistence of increased levels may be related to activation of the immune system [61, 66] and metabolic side effects of immunosuppressive agents (calcineurin inhibitors and corticosteroids) [67, 68].

### 4.3. Advanced Glycation End Products (AGEs)

Advanced glycation end products (AGEs) are a heterogeneous group of compounds derived from the nonenzymatic glycation of proteins, lipids, and nuclear acids through a complex sequence of reactions referred to as the Maillard reaction [69]. N-Carboxymethyllysine (CML), pentosidine, and hydroimidazolone are among the best characterized of at least 20 different types of AGEs and serve as markers of AGE accumulation in tissues [70, 71]. Interactions between AGEs, their receptors, and advanced glycation end product receptors (RAGE) trigger a cascade of various events leading to endothelial dysfunction, arterial stiffness, immune system dysregulation, and atherosclerosis progression [72].

Accumulation of AGEs in CKD patients is a result of oxidative stress and inflammation and comes from external sources such as diet and cigarette smoke [72, 73]. AGEs are metabolized and removed by the kidneys. They are filtered through the glomerulus and reabsorbed by renal proximal tubules, and both processes are complex and variable [74, 75]. The kidneys are also a place of accumulation and AGE-related organ damage [76], and progressive retention of AGEs occurring with declining renal function creates a vicious cycle of kidney damage and accelerated decline in renal function. Therefore, in CKD, increased levels of AGEs may be seen as a result of impaired clearance and enhanced formation in response to oxidative stress and/or carbonyl stress. Serum AGE levels correlate inversely with eGFR, and they appear to be predictive for the development of reduced glomerular filtration rate [77–79]. In Semba et al.'s [79] study, the increase in AGE levels was evident from CKD stage 3.

Kidney transplantation is the most effective therapy to reduce elevated levels of AGEs. Nevertheless, in renal transplant recipients, AGEs remain higher than in normal subjects and disproportionally higher than the GFR alone would imply [80, 81]. It suggests that other factors may influence the formation of AGEs. Factors contributing to increased accumulation of AGEs, and at the same time, the risk of chronic graft dysfunction, include the dialysis vintage before transplantation, donor age, and primary graft function. Closely related to the formation of AGEs is the state of increased oxidative stress typical of kidney transplant recipients, the determinants of which may be diabetes mellitus, ischemic/reperfusion injury, immunosuppressants, and renal failure [81–83]. In Shahbazian et al.'s [83] study, the levels of AGEs were significantly increased in renal transplant patients with measured GFR below 30 ml/min, and a significant association between the levels of AGEs and measured GFR was found.

### 4.4. Phosphate, Klotho, and FGF23

Abnormalities of mineral metabolism are universal complications of CKD associated with accelerated atherosclerosis and vascular calcification and correlated with increased mortality across all stages of CKD, independent of traditional risk factors [84–86]. The levels of serum phosphate, calcium, and parathyroid hormone are influenced by *α*-Klotho, FGF23, 1,25-dihydroxyvitamin D, diet, and medications, interacting with each other in complicated ways.

*α*-Klotho not only functions as one of the regulators of mineral homeostasis but also exerts pleiotropic biological effects including antioxidative stress, antiapoptosis, and antiaging [87, 88]. *α*-Klotho is expressed in multiple tissues; however, the strongest expression is in the kidney [89]. Kidney injury and subsequent renal impairment will result in the decrease of *α*-Klotho production. It has been shown that serum *α*-Klotho starts to decline in stage 2 CKD, and urinary *α*-Klotho even earlier, in stage 1 CKD [90], and for each 1 ml/min/1.73m^2^ eGFR decrease, an adjusted mean decrease of 3.2 pg/ml of serum *α*-Klotho was revealed [91]. Furthermore, pbUTs inhibit *α*-Klotho expression [92]. Clinical and experimental studies have shown that the decrease of *α*-Klotho is positively associated with eGFR [87, 93, 94].

Fibroblast growth factor 23 (FGF23) is a bone-derived phosphatonin, which acts in the kidney to induce urinary phosphate excretion and suppress 1,25-dihydroxyvitamin D synthesis, in the presence of FGF receptor 1 (FGFR1) and its coreceptor *α*-Klotho [95, 96]. It has been also shown that FGF23 has a deleterious effect on vascular function—endothelial dysfunction, atherosclerosis, left ventricular hypertrophy, and increased risk of major cardiovascular events [97–99].

The increase in FGF23 is a compensatory reaction in response to decreased expression of transmembrane *α*-Klotho to maintain mineral homeostasis, so in early stages of CKD, serum phosphates are not elevated. In turn, increased levels of FGF23 decrease *α*-Klotho expression, and finally, dietary phosphorus overload cannot be compensated and contributes to overt hyperphosphatemia in advanced stages of CKD [96]. FGF23 levels increase progressively in early stages of CKD. It is suggested that renal injury itself may be an initial stimulus for FGF23 secretion [100]. In Isakova et al.'s [101] study, 33% of participants with eGFR ≥ 70 ml/min and 85% with eGFR 30-60 ml/min had elevated levels of FGF23, and in a dialyzed patient, serum levels of FGF23 are extremely high reaching levels that can be 1000-fold above the normal range [101]. Moreover, a strong correlation between eGFR and FGF23 was revealed [91, 101].

Close to 90% of patients with 3-4 CKD stage have normal phosphate levels, and with the progressive loss of functional nephrons, the compensatory mechanism is overwhelmed, and most patients with ESRD have overt hyperphosphatemia. Hyperphosphatemia is considered to be a risk factor for cardiovascular and all-cause mortality, and for each 1 mg/dl increase in serum phosphate, the risk of death is increased by 18-20% [102, 103].

The data on levels of *α*-Klotho and FGF23 in transplant recipients are scarce, and sometimes inconsistent. During the first week after kidney transplantation, the decrease in serum levels of *α*-Klotho was noted [104, 105]. This initial decline is probably multifactorial and may be a response to trauma and tissue injury, transient kidney tubular dysfunction, and the impact of immunosuppression therapy [104, 106]. In the consecutive weeks, the gradual increase of *α*-Klotho was observed with the highest levels exhibited at 52 weeks posttransplantation and compared with pretransplant levels [104]. However, no association between serum *α*-Klotho levels and kidney function has not been demonstrated in Tan et al.'s study, as well as in three other cross-sectional studies [107–109].

FGF23 levels decline in the postrenal transplantation period; however, they remain higher than in CKD patients matched for eGFR [104, 110–113]. Further reductions in FGF23 levels are observed over longer follow-up, approximating normal levels 1–3 years after transplantation [110].

In up to 90% of transplant recipients, mild to moderate hypophosphatemia is present. Phosphate levels remain low for longer than in patients with CKD matched for the eGFR [114]. Kidney function does not play a crucial role in posttransplant hypophosphatemia but persistently high levels of FGF23 and PTH [113, 115].

### 4.5. Oxidative Stress: The Impact of Kidney Function

Oxidative stress (OS) is defined as a state of imbalance between excessive prooxidant activities relative to antioxidant defense mechanisms. Oxidative stress leads to metabolic dysregulation and oxidation of lipids, proteins, and nucleic acids and oxidative damage in cells, tissues, and organs caused by ROS and reactive nitrogen species (RNS) [116, 117]. OS is frequently observed in CKD patients; contributes to inflammation, endothelial dysfunction, risk of atherosclerosis, and progression of CKD [118]; and is considered one of the nontraditional risk factors for cardiovascular and all-cause mortality [119, 120]. OS through generation of uremic toxins enhanced intestinal permeability to endotoxins and alteration in nitrogen handling [121–123]:

- Accumulation of AGEs activating transcription factors (NF-*κ*B, AP1, and SP1) executed via RAGE, and activation of NADPH oxidases (NOXs) which directly generate free radicals [124, 125]
- Inflammation, which is spliced with OS—inflammatory cells stimulate the release of reactive species, and oxidized end products stimulate phagocytic cells to release inflammatory cytokines and ROS creating a positive feedback loop; the leading feature is the two-way interplay between NOX, NF-*κ*B, inflammasomes, and phagocytic cells [126, 127]
- Dialysis increases the state of oxidative stress, and the involved mechanisms include the use of bioincompatible membranes and fluids, contamination of dialysate with bacterial endotoxins, and occult infections [128–130]

The imbalance in oxidant-antioxidant status begins early in the course of CKD. It was shown that increased levels of NADPH-generated ROS and lower levels of the antioxidant enzymes can be revealed in patients with 1 and 2 CKD stage [124, 131–133]. Progressive loss of renal function results in increased oxidative stress and inflammation, and a positive correlation between advancing stage of CKD and increasing oxidative stress has been demonstrated [134–137]. The inverse relationship between eGFR and markers of oxidative stress was revealed in several studies [136–138], but in some, the correlation was at least weak [139]. It is possible that this difference may be a result of biomarkers used and studied populations.

Successful kidney transplantation leads to a reduction in metabolic abnormalities and significant improvement in OS-related markers. Normalization of graft function seems to be a key factor in the restoration to near-normal levels of OS biomarkers. Despite the fact that surgical procedure of kidney transplantation and ischemic injury during the procurement and organ transfer cause an oxidative burst, the improvement of OS can start immediately after transplantation [140]. Sudden cessation of blood flow during organ donation cause ischemic/hypoxic injury [141, 142]. Cold storage promotes ROS production via mitochondrial dysfunction. ROS react with other molecules, leading to oxidative damage of proteins, nucleic acids, and lipid peroxidation and contribute to cell apoptosis [143–145]. The reperfusion stage, during which blood flow is restored, leads to a burst of ROS and is regarded as the final stage of ischemic injury [141–146]. OS in kidney transplant recipients may be, at least in part, caused by the immunosuppressive therapy. Most of the currently used immunosuppressive medications, such as corticosteroids and calcineurin inhibitors (cyclosporine A and tacrolimus), may contribute to the increased OS. The prooxidant activities of tacrolimus and cyclosporine A, the indispensable parts of immunosuppressive, have been studied. Some studies reported that increased levels of malondialdehyde are a consequence of immunosuppressive therapy and that OS is induced mostly by cyclosporine A [147, 148]. Other studies, however, have not confirmed these findings [140, 149, 150]. Other factors, such as opportunistic infection or immune response to allograft, may also trigger OS in kidney transplant recipients [151].

CKD-associated OS in pretransplant phase, reperfusion injury, and increased immunosuppression are considered the key factors of continual OS during the early phase of transplantation [151–153]. Over the next days, the improvement of antioxidant status is observed along with the restoration of kidney function, reduction in metabolic abnormalities, and decrease in OS [152, 154–157]. Some controversies regarding changes in enzymatic and nonenzymatic antioxidants as well as OS biomarkers may probably arise from the study design and different observation periods. In some studies, the increase in antioxidant systems and decrease in OS were observed already in the early posttransplant period [154–157]. In other studies, during the first 2 weeks, a significant increase in lipid peroxidation [140, 151, 158] and decrease in erythrocyte glutathione or superoxide dismutase activities were observed [159, 160]; however, in longer observation (28-day posttransplantation), the decrease in lipid peroxidation along with antioxidant system activities was revealed [140, 151, 158]. The levels of advanced oxidation protein products (AOPPs) decrease immediately after transplantation. As long as reduction in the first day may be explained by blood loss during surgery, the decrease in subsequent days confirms that successful kidney transplantation provides efficient elimination of generated ROS [154–157, 161, 162].

Most studies have shown that reestablishment of kidney function improves the OS over few weeks after transplantation [140, 154–162]. Time-dependent changes in OS biomarkers are associated with improvement in kidney function, and the levels of AOPPs and low molecular AGEs correlate inversely with creatinine clearance [140, 151, 154, 155, 157]. Normalization of graft function may restore to near-normal levels of OS biomarkers, regardless of immunosuppression used; however, achieving any level of kidney function will decrease OS level [150, 163, 164]. The reduction in OS after transplantation may be also a prognostic factor of short- and long-term graft function and CVD in this patient population [163, 165].

### 4.6. Implications of Uremic Toxins and Oxidative Stress to Atherosclerosis

In CKD, endothelial dysfunction and atherosclerosis are almost universal, as well as cardiovascular complications as first reported by Lindner et al. [166], who drew attention to the excessive incidence of atherosclerotic cardiovascular mortality in dialyzed patients. Various CKD-specific factors and processes are involved in endothelial dysfunction in CKD as presented in Figure 1. It is characterized by proinflammatory and prothrombotic endothelial phenotype, structural damage, impaired capabilities of protective and repair mechanisms, and increased oxidative stress. Uremic toxins, when in high concentrations in the bloodstream, play an important role in endothelial dysfunction, which in turn contributes to the pathogenesis of cardiovascular diseases, such as atherosclerosis and thrombotic events [35–39]. Each toxin can play its own role in vascular dysfunction, as presented in Table 1; however, its accumulation and coexistence potentiate the deleterious effects.

Inflammation is considered one of the main mechanisms of atherosclerosis, and CKD is a state of systemic inflammation [34, 167, 168]. It depends both on the increased synthesis and decreased elimination of mediators of inflammation, and multiple cytokines are involved in the genesis of this proinflammatory milieu in CKD [169]. Uremic toxins induce inflammation in endothelial cells (ECs) and stimulate the cross-talk between ECs and macrophages [14, 35–37]. In the response to the injury, the concentration of cytokines is increased leading to the activation of endothelial, resident vascular cells, and circulating monocytes [8, 11, 36–38]. Uremic toxins (pbUTs, phosphates, and FGF23) increase the expression of adhesion molecules (E-selectin, P-selectin, ICAM-1, and VCAM-1) promoting the infiltration of monocytes and macrophages in the activated endothelium [11, 13, 15, 16, 20, 35, 37].

Uremic toxins promote the production of ROS and decrease antioxidant defenses, resulting in oxidative stress [10, 21, 27, 118, 119, 127]. ROS activate transcription factors leading to the expression of inflammatory cytokines, as well as causing mitochondrial dysfunction, inducing cell death [117, 126, 170]. At the same time, uremic toxins inhibit late-stage autophagy, making cells more sensitive to oxidative stress and contributing to endothelial dysfunction. It may lead to atherosclerosis and arterial aging [171, 172].

Uremic toxins contribute to structural damage of ECs resulting in increased endothelial permeability. In vitro studies demonstrated that uremic toxins (pbUTs and phosphate) induce cytoskeletal remodeling, resulting in the changes in EC morphology, and lead to the rupture of cell-cell junctions damaging endothelial barrier and contributing to increased permeability [173–175]. Endothelial damage results in a release of microparticles and specific miRNAs that may further promote vascular damage. Endothelial microparticles (EMPs) are important in intracellular communication. Uremic toxins (pbUTs and phosphate) induce the formation of EMPs from endothelial cells [19, 176–178]. Uremic toxins induced EMPs show different activities: they have an antiangiogenic effect on endothelial progenitor cells impairing endothelium repair process [179], have procoagulant activity due to the production of factor Xa and tissue factor (TF) [179], enhance the proliferation of VSMC contributing to neointimal hyperplasia [180], and finally increase osteocalcin expression in ECs, VSMC, and fibroblast, which indicates vascular calcification [181]. MicroRNAs participate in the regulation of EC function modulating angiogenesis and immune response [182]. Uremic toxins upregulate miRNAs causing suppression of expression of genes responsible for endothelial homeostasis and thus contributing to EC dysfunction and apoptosis [182, 183].

Uremic toxins also cause a reduction in the number and function of endothelial progenitor cells. Protein-bound UTs and AGEs suppress the expression of transcription factors, SIRT1 and KLF2, responsible for the maintenance of endothelial homeostasis, inhibiting oxidative stress and cell senescence [182, 184, 185].

Uremic toxins contribute to the prothrombotic state of endothelium leading to an increased risk of thrombotic events, such as thromboembolism and ischemia. Furthermore, in CKD, the processes of coagulation and fibrinolysis are impaired with increased levels of tissue factor (TF), von Willebrand factor (vWF), thrombomodulin, factor VIII, and D-dimer [186]. In vitro studies demonstrated that uremic toxins (IxS and IAA) increase the expression of TF and production of factor Xa indicating endothelial activation and procoagulant activity [179]. Uremic toxins (phosphate, IxS, and ADMA) also decrease the production and/or bioavailability of NO which acts as an inhibitor of platelet adhesion and aggregation [187–189].

Endothelial cell integrity and function are critical to the prevention of atherosclerosis; therefore, dysfunction of endothelium is critical in the development of vascular dysfunction and progression of CVD. Nevertheless, uremic toxins participate in atherosclerosis development in many steps. They influence proliferation, migration, calcification, and senescence of VSMC [9–11, 16, 20, 23, 26, 34, 35]. They also induce chronic activation of leukocytes (monocytes and neutrophils), stimulate the leukocyte-endothelial interactions, and promote vascular wall infiltration by inflammatory cells [12–15, 34, 37, 167–169]. And finally, uremic toxins participate in the formation of atherosclerotic plaque and its rupture [1, 33–35].

## 5. Final Considerations

It would be worth to mention that AKI contributes to the initiation and progression of CKD, and vice versa CKD predisposes to AKI [190–192]. AKI and CKD are interconnected syndromes. The accumulating data from basic and clinical research indicates that renal hypoxia is associated with CKD, AKI to CKD continuum, and AKI on top of CKD. Tubulointerstitial hypoxia is a key player in the pathophysiology of CKD and AKI to CKD transition [193–198]. Capillary rarefaction after AKI episode results in tubulointerstitial fibrosis, and damaged tubular epithelial cells that fail to redifferentiate may contribute to capillary rarefaction and thus aggravating hypoxia [193, 194, 199]. Moreover, hypoxia induces diverse epigenetic changes such as chromosome conformation, DNA methylation, or histone modification [199]. The mechanisms involved in the susceptibility of AKI and impairment of recovery from AKI in CKD patients remain largely unexplained. Multiple mechanisms at epigenetic, signaling, cellular, and tissue levels may be involved [200–202]. Briefly, oxidative stress is a key mechanism in the pathogenesis and progression of CKD and impaired renal regeneration after AKI episodes. Therapeutic strategies targeting hypoxia have been shown to be effective in blocking the progression to CKD and possibly AKI protection [192, 193, 199].

In CKD, the retention of a variety of metabolites, due to a decrease in their renal clearance and/or a rise in their synthesis, is found. These compounds could be small and water soluble, lipophilic and/or protein bound, or larger and in the middle-molecule range. Several solutes have been shown to exert biological activity, on cells and metabolic processes, leading to uremic syndrome. Moreover, dietary protein breakdown, alternative sources such as environmental contact, food additives, natural stimulants (coffee and tea), herbal medicines, or addiction to psychedelic drugs, may also play a role in uremic toxicity. Slowing of the progression of CKD thereby preservation of kidney function is crucial in the removal of uremic toxins. Successful kidney transplantation with good graft function offers the best possibility to lower the levels of uremic toxins. In addition, uptake of uremic toxins in the intestine could be decreased by influencing dietary uptake, oral administration of sorbents, or administration of prebiotics or probiotics influencing intestinal flora. Moreover, changing the source of protein intake from animal-based to plant-based diet may also reduce intestinal production of uremic toxins. Other therapeutic intervention includes administration of drugs countering the biological impact of uremic solutes such as angiotensin-converting enzyme inhibitors (ACEi) which neutralize Ca influx due to SDMA [203]. Moreover, the IxS level can be decreased by rising sulfotransferase activity, responsible for indole sulfation [204].

In addition, the development of therapeutic strategies to raise *α*-Klotho and lower phosphate, FGF23, and other uremic toxins is of great importance as they may contribute to the decline in cardiovascular morbidity and mortality in CKD and after kidney transplantation.

## Figures and Tables

**Table 1:** The mechanisms of action of selected uremic toxin impact on cardiovascular damage.

Protein-bound uremic toxins (para-cresyl sulfate, indoxyl sulfate)	Impairment of vascular reactivity and induction of vascular remodeling; induction of oxidative stress; stimulation of proinflammatory responses in vascular cells and macrophages; promotion of adhesion molecule expression; stimulation of the cross-talk between macrophages and endothelial cells promoting vascular wall infiltration by inflammatory cells [9–15]

Phosphate	Increase in contraction and decrease in endothelium-dependent relaxation of the vessels; increased production of ROS in VSMC and in endothelial cells via NADPH oxidase activation; induction of EMP shedding resulting in the impairment of endothelial cells with thrombotic, inflammatory, and antiangiogenic properties [16–19]

Klotho and FGF23	Arterial stiffness via a downregulation of SIRT1 expression in endothelial and smooth muscle cells; induction of an increase in oxidative stress, reduced NO production, induced the expression of cell adhesion molecules [20–23]

ADMA	Reduction of NO production; induction of oxidative stress and acceleration of the senescence of endothelial cells [24–27]

AGEs	Osteogenic-like differentiation of SMCs and subsequent calcification; promotion of inflammation and oxidative stress via activation of NADPH oxidase, upregulation of adhesion molecule expression; induction of vascular contraction by modulating ET-1 expression; induction of endothelial cell apoptosis and impairment of endothelial progenitor cell survival, differentiation, and function [28–32]

## Data Availability

There are no supporting data.

## Abbreviations

ACEi: Angiotensin-converting enzyme inhibitor
ADMA: Asymmetric dimethylarginine
AGEs: Advanced glycation end products
AGTX2: Alanine glyoxylate aminotransferase 2
AKI: Acute kidney injury
ANP: Atrial natriuretic peptide
AP1: Activator protein 1
CKD: Chronic kidney disease
CKD-MBD: CKD-mineral bone disorder
CML: N-Carboxymethyllysine
CVD: Cardiovascular disease
DDAH: Dimethylarginine dimethylaminohydrolase
eGFR: Estimated glomerular filtration rate
ECs: Endothelial cells
EMP: Endothelial microparticles
ESRD: End-stage renal disease
ET-1: Endothelin 1
FGF23: Fibroblast growth factor 23
FGFR1: Fibroblast growth factor receptor 1
IAA: Indole-3-acetic acid
ICAM-1: Intercellular adhesion molecule-1
IL-6: Interleukin 6
IL-18: Interleukin 18
IL-1*β*: Interleukin 1*β*
IxS: Indoxyl sulfate
KLF2: Krüppel-like factor2
NADPH: Nicotinamide adenine dinucleotide phosphate
NF-*κ*B: Nuclear factor kappa-light-chain-enhancer of activated B cells
NO: Nitric oxide
NOX: NADPH oxidase
PAI-1: Inhibitor of tissue plasminogen activator
p-CS: p-Cresyl sulfate
p-CG: p-Cresyl glucuronide
PTH: Parathyroid hormone
RAGE: Advanced glycation end product receptor
RNS: Reactive nitrogen species
ROS: Reactive oxygen species
SDMA: Symmetric dimethylarginine
SIRT1: Sirtuin 1
SMC: Smooth muscle cell
SP1: Specificity protein 1
TF: Tissue factor
TFPI: Tissue factor pathway inhibitor
TMAO: Trimethylamine-N-oxide
TNF*α*: Tumor necrosis factor *α*
t-PA: Tissue plasminogen activator
VCAM-1: Vascular adhesion molecule-1
VEGF: Vascular endothelial cell growth factor
vWF: von Willebrand factor
VSMC: Vascular smooth muscle cells.
